# Distributions of endocrine cell clusters during porcine pancreatic development

**DOI:** 10.1371/journal.pone.0216254

**Published:** 2019-05-10

**Authors:** Masaki Nagaya, Asuka Hayashi, Kazuaki Nakano, Michiyo Honda, Koki Hasegawa, Kazutoshi Okamoto, Shiori Itazaki, Hitomi Matsunari, Masahito Watanabe, Kazuhiro Umeyama, Hiroshi Nagashima

**Affiliations:** 1 Meiji University International Institute for Bio-Resource Research, Kawasaki, Japan; 2 Department of Immunology, St. Marianna University School of Medicine, Miyamae-ku, Kawasaki, Japan; 3 Laboratory of Developmental Engineering, Department of Life Sciences, School of Agriculture, Meiji University, Kawasaki, Japan; International University of Health and Welfare School of Medicine, JAPAN

## Abstract

**Background:**

Pancreatic islet xenotransplantation is a potential treatment for diabetes mellitus, and porcine pancreas may provide a readily available source of islets. Islets in juvenile pigs are smaller than those in young adult pigs, but the insulin content is very similar. In addition, as juvenile pigs are more easily reared in uncontaminated conditions, many researchers have conducted studies using pancreatic islets from juvenile pigs. We aimed to analyze the distributions of endocrine cell clusters by comprehensively evaluating juvenile porcine pancreatic development and to propose an appropriate age at which islets could be isolated from the juvenile porcine pancreas.

**Methods:**

Splenic (SL) and duodenal lobe (DL) samples were collected from the pancreases of pigs aged 0–180 days (n = 3/day after birth). The chronological changes in endocrine cell clustering were analyzed in relation to morphological changes, cell characterization, numbers, islet areas, and gene expression.

**Results:**

In juvenile pigs aged 0–21 days, the pancreas contained numerous endocrine cells, and compact islets appeared from 21 days of age. Well-defined small islets were seen at 28 days of age, and the clusters were denser in the SL than in the DL. At 35 days of age, the islets were morphologically similar to those observed at 180 days of age, and the greater number of islets was similar to that seen at 90 days of age. The differences in the islets’ cytoarchitecture between the lobes were negligible. The expression of β-cell-related genes was higher in the juvenile pancreas than in the adult pancreas, and the expression of *neurogenin-3* decreased dramatically over time.

**Conclusions:**

These findings may have implications for attempts to refine the most appropriate age for islet isolation from porcine donors. Focusing on porcine pancreatic islets isolated at around 35 days after birth may offer benefits regarding their xenotransplantation potential.

## Introduction

Diabetes causes several complications in patients, which burden health care systems. Allogeneic islet transplantation could improve physiological glucose metabolism [1]. However, the number of available human donor organs has not kept pace with the growing transplant candidate waiting lists, and a significant number of these patients die without receiving transplants [2]. This allotransplantation issue has stimulated research on the induction of insulin-producing cells and bioengineering. An alternate approach is the use of living cells, tissues, or organs from another species as donors. The concept of cross-species transplantation is known as xenotransplantation, and the transplanted cells, tissues, or organs are called xenografts. Xenotransplantation is an attractive option to overcome the issue of donor shortage [3, 4].

Pigs have been selected as the most suitable potential source of organs for xenotransplantation, because there are many similarities between humans and pigs, including similarities related to physiology and anatomy [5, 6]. Additionally, porcine and human insulin differ by only one amino acid, and porcine insulin has been used for several decades in clinics. Pigs are widely available in many countries, are easily bred, and are known to have large litters. Additionally, ethical issues associated with the slaughter of nonhuman primates do not exist. Moreover, ethical and regulatory frameworks for islet xenotransplantation are present in several countries, and now, there is a greater awareness of the importance of developing an internationally harmonized ethical and regulatory framework [7]. Thus, pig-to-human xenotransplantation offers a potential bridge to the growing disparity between graft requirement among patients with diabetes and graft availability [3, 4]. Indeed, islet transplantation from porcine pancreas was performed for the first time in the 1990s [8]. The International Xenotransplantation Association (IXA) launched a consensus statement in 2009 on conditions for safely undertaking clinical trials of porcine islet products in patients with type 1 diabetes [9]. This statement covers the key aspects of ethical requirements. Recently, clinical porcine islet xenotransplantation was restarted under comprehensive regulations in New Zealand [10].

However, several factors impede the clinical translation of this promising therapeutic modality. First, rearing pigs in a gnotobiotic environment is costly and technically difficult, and considerable islet losses occur during cell isolation and tissue culture because of fragmentation [11]. Second, zoonotic diseases, for example, diseases involving porcine endogenous retroviruses (PERVs), could spread from pig donors to human recipients after transplantation. Third, islets from young adult and adult pigs have not been used in clinical trials so far [6].

Many researchers have conducted studies using juvenile porcine pancreatic islets, because juvenile pigs are more easily reared in uncontaminated conditions before use. Lamb et al. reported that juvenile pigs produce the highest and most consistent yields of extremely pure, viable islets that respond satisfactorily to glucose challenges in vitro [12]. Indeed, following encapsulated neonatal porcine islet transplantation, some patients showed reductions in the frequencies of unaware hypoglycemic events [10]. The consensus from the IXA states that hypoglycemia unawareness could justify islet xenotransplantation [13]. A phase 1/2a trial involving xenotransplantation of encapsulated neonatal porcine islets is being conducted in New Zealand under the regulatory framework of the IXA consensus statement [10, 14, 15]. The transmission of PERVs during this clinical trial of porcine islet transplantation has not been confirmed [10, 14– 16], and the risk of PERV-related complications is considered to be low [17].

Regarding the use of juvenile porcine islets as donor xenografts, the morphology, size, number, and distribution of the islets might influence the xenotransplantation results. Although studies involving rodent and human pancreases have substantially improved our understanding of the molecular processes that regulate pancreatic development [18– 32], the transcription factor cascades that control porcine pancreatic endocrine cell differentiation and function remain largely unknown. Hence, the present study was conducted to explore the appropriate age at which islets could be isolated from porcine donors for future xenotransplantation by characterizing the developmental changes that occur in porcine pancreas from 0 days to 180 days of age. Furthermore, we investigated the molecular events that occur during pancreatic development by evaluating transcription factor genes known to be involved in pancreatic development.

## Materials and methods

### Animal care

All of the animal experiments in this study were approved by Meiji University’s Institutional Animal Care and Use Committee (IACUC-13-0019). We used 24 crossbred Large White/Landrace × Duroc pigs of either sex. All animals were housed and maintained in accordance with IACUC guidelines. All animal care and experimental procedures were performed in accordance with the regulations contained in the Japanese Act on Welfare and Management of Animals. Pigs were housed in a temperature-controlled room, had free access to water and were provided with growth-stage appropriate commercial feed (Chubushiryo Co., Ltd. Nagoya, Japan) and were observed on a daily basis by animal husbandry personnel under the supervision of an attending veterinarian. The health of all pigs was assessed at feeding (08:00 and 17:00).

### Surgical procedure

**Fig 1:**
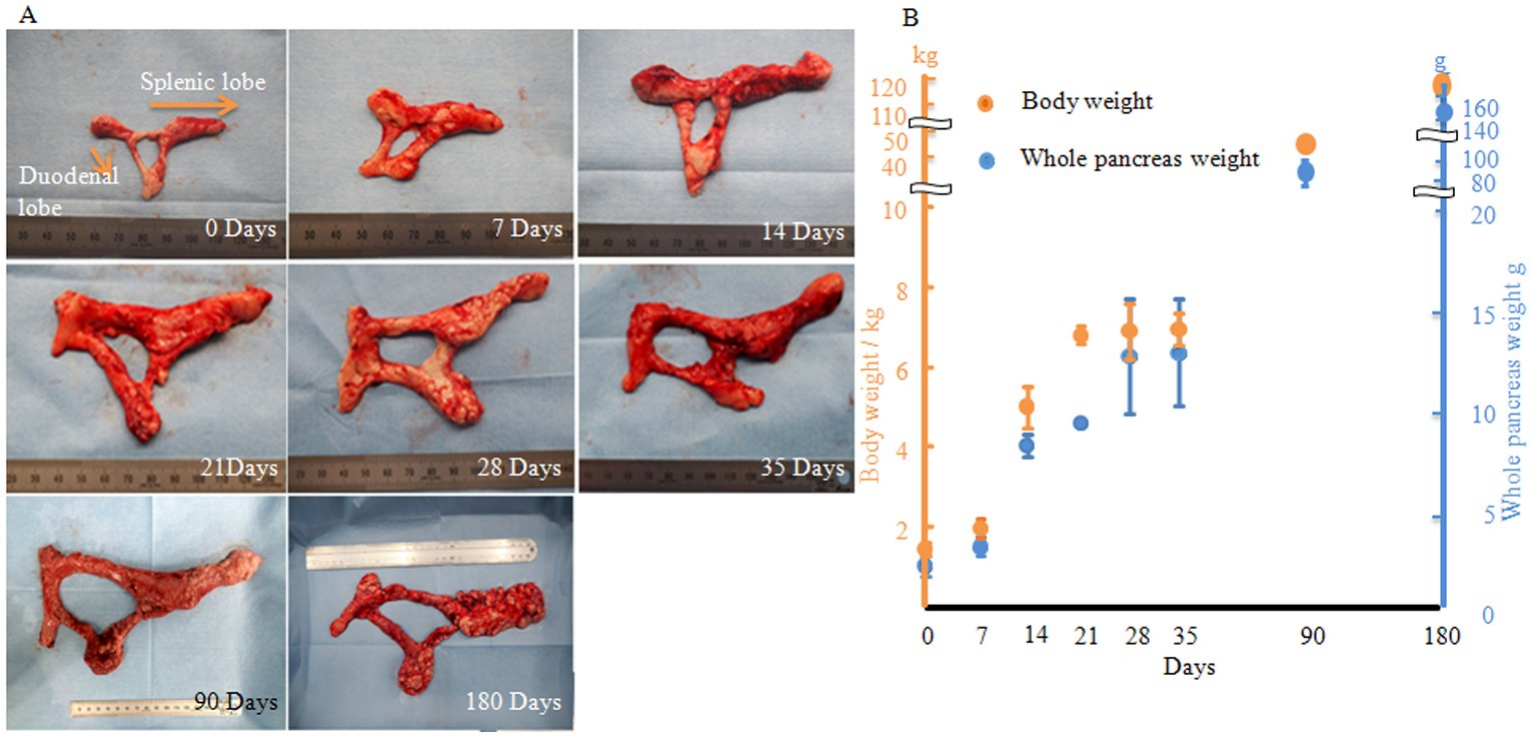
Correlation between the whole pancreas and body weights during porcine pancreatic development. (A) Developmental changes in the whole pancreas. The panels show changes on each day, from 0 to 180 days. (B) Body and whole pancreas weights at specified times. Both the body and whole pancreas weights presented are the means ± standard deviations (n = 3/day after birth). The orange and blue bars indicate the body and whole pancreas weights, respectively. The time after birth is represented as days. SD = standard deviation.

The pancreases were harvested from pre-weaned juvenile pigs aged 0–21 days, weaned juvenile pigs aged 28 and 35 days, and young adult pigs aged 90 and 180 days that were fed solids. To obtain the pancreases, the pigs were anesthetized using intramuscular injections of ketamine (11 mg/kg) (Fujita Pharmaceutical Co., Ltd., Tokyo, Japan), and maintained under isoflurane anesthesia (DS Pharma animal Health Co., Ltd., Osaka, Japan). The animals were then placed in the supine position, and the surgical area was disinfected with povidone iodine (Meiji Seika Pharma Co., Ltd., Tokyo, Japan). A midline abdominal incision was made under sterile conditions, the pancreas was gently separated from the surrounding tissues, and the whole pancreas was excised (Fig 1A). During surgery, Ringer’s solution was administered at 10 mL/kg per hour. The animals’ body weights and whole pancreas weights were recorded. Two pancreatic tissue samples that measured 1.0 cm were collected from the splenic lobe (SL) and the duodenal lobe (DL) for the analyses. At the time of removal of pancreas, the pigs are euthanized by intravenous bolus injection of potassium chloride (1–2 mmol/kg).

### Immunohistochemical analysis

One tissue sample was immediately fixed in 4% paraformaldehyde, embedded in paraffin, and sectioned at 4 μm. Previously described immunohistochemical methods were used [33]. Briefly, after deparaffinization and blocking, the sections were incubated with diluted primary antibodies overnight at 4°C. The primary antibodies used were guinea pig anti-insulin (Linco Research Immunoassay, St. Charles, MO, USA) and mouse anti-glucagon (Sigma Aldrich Japan, Tokyo, Japan). The secondary antibodies comprised Alexa488-conjugated and Alexa594-conjugated antibodies (Molecular Probes, Eugene, OR, USA). The cells’ nuclei were counterstained with Hoechst 33342. The negative controls comprised sections that were incubated with the secondary antibodies only, and the positive controls comprised stained islets. Sections from each location were stained with hematoxylin and eosin and incubated with each antibody of interest. The sections were examined under a confocal microscope (BZ-X700; Keyence, Osaka, Japan), and software was used for the data analysis (BZ-H3A; Keyence).

### Definition of β-cells, islet cell clusters, and islets

β-cells were defined as single or pairs of insulin-positive cells. Islet cell clusters (ICCs) were defined as cell aggregates of various sizes that were not condensed, but consisted of glucagon- and insulin-positive cells. Islets were defined as dense cell aggregates that comprised glucagon- and insulin-positive cells with well-defined edges.

### Quantifying the β-cells, islet cell clusters, and islets during porcine pancreatic development

The β-cells, ICCs, and islets were analyzed using a computer system (Cosmos32 Library, Tokyo, Japan) to determine their numbers, and to determine the islets’ diameters, and areas. To evaluate the distributions of the β-cells, ICCs, and islets, we counted them in 3 randomly selected areas measuring 1.65 mm^2^, and we examined 9 samples of each pancreatic lobe from 3 different pigs of the same age. The mean values for each lobe were calculated for each day assessed. Porcine islets can be round, oval, triangular, dumbbell-shaped, and all shapes in-between [5]. To calculate the islets’ areas and to overcome errors caused by irregularly shaped structures, the islets’ circumferences were demarcated, and software was used for the data analysis.

### Ribonucleic acid isolation, complementary deoxyribonucleic acid synthesis, and the quantitative real-time polymerase chain reaction

To investigate the molecular events that occur during pancreatic development, seven transcription factor genes known to be involved pancreatic development [18– 32] were selected and evaluated in this study. The gene expression profiles were determined using the quantitative real-time polymerase chain reaction (qPCR). After isolating total ribonucleic acid (RNA) using the RNAspin Mini Kit (GE Healthcare, Little Chalfont, England), it was reverse-transcribed into complementary deoxyribonucleic acid (cDNA) using the Transcriptor First Strand cDNA Synthesis Kit (Roche Diagnostics, Rotkreuz, Switzerland), according to the manufacturers’ instructions. The qPCRs were performed using the Light Cycler 480 SYBR Green I Master Kit (Roche Diagnostics) and a Light Cycler System (Roche Diagnostics), according to the manufacturer’s instructions. The primers used (Sigma Aldrich Japan, Tokyo, Japan) are shown in Table 1. For the qPCRs, 20 ng of cDNA was used, and the messenger RNA (mRNA) levels were determined from the average of reactions performed in triplicate. The following conditions were used for the qPCRs: 40 cycles of 30 s at 94°C, 90 s at 60°C, and 90 s at 72°C. The mRNA levels were normalized to those of *actin gamma 1(ACTG1)* and the normalized values were compared with those from a porcine pancreas aged 180 days. To determine *neurogenin-3* (*NGN3*) expression, day 0 samples were used for the normalization, and the expression levels were determined as the average of reactions performed in triplicate.

**Table 1:** Characteristics of the target genes evaluated using the real-time polymerase chain reaction.

Symbol	Gene name	RefSeq ID	PCR size (bp)
*PDX1*	*pancreatic and duodenal homeobox 1*	NM_001141984	72
*NGN3*	*neurogenin-3*	KP796255	80
*NEUROD1*	*neuronal differentiation 1*	XM_005654180	95
*Nkx 6–1*	*NK6 homeobox 1*	XM_021101796	89
*MAFA*	*v-maf avian musculoaponeurotic fibrosarcoma oncogene homolog A*	XM_003354965	100
*GLUT2*	*glucose transporter type 2* *(solute carrier family 2 member 2 (SLC2A2))*	NM_001097417	105
*INS*	*insulin*	NM_001109772	140
*ACTG1*	actin gamma 1	XM_003357928	109

PCR = polymerase chain reaction; Ref SeqID = Reference Sequence Identification.

### Statistical analyses

The data were averaged and expressed as the means (standard deviations) or the means (standard errors of the means). To compare the groups, the unpaired Student’s t-test, repeated-measures one-way analysis of variance, and Fisher’s protected least significant difference test were used. A value of *P* < 0.05 was considered statistically significant.

## Results

### Correlations between whole body and pancreas weights during porcine pancreatic development

The body and whole pancreas weights increased consistently with the developmental changes (Fig 1B). These weights increased in parallel during the developmental stages, almost plateaued at 28 days, and increased again. At 28 days, the pancreases were firm (Fig 1A).

### Architectural changes in the porcine pancreas during development

At 14 days of age, lobular structures were present in the pancreas, and the duct- and vessel-like structures were thin and immature (S1A and S1A’ Fig). At 21 days of age, the duct- and vessel-like structures resembled those at 14 days of age (S1A, S1A’, S1B and S1B’ Fig). By 28 days of age, significant changes in the tissue architecture were noted. The circumferences of the duct- and vessel-like structures had thickened as a consequence of connective tissue surrounding these structures to resemble the adult configuration (S1C, S1C’, S1D and S1D’ Fig).

### Morphological changes in endocrine cell clustering during porcine pancreatic development

**Fig 2:**
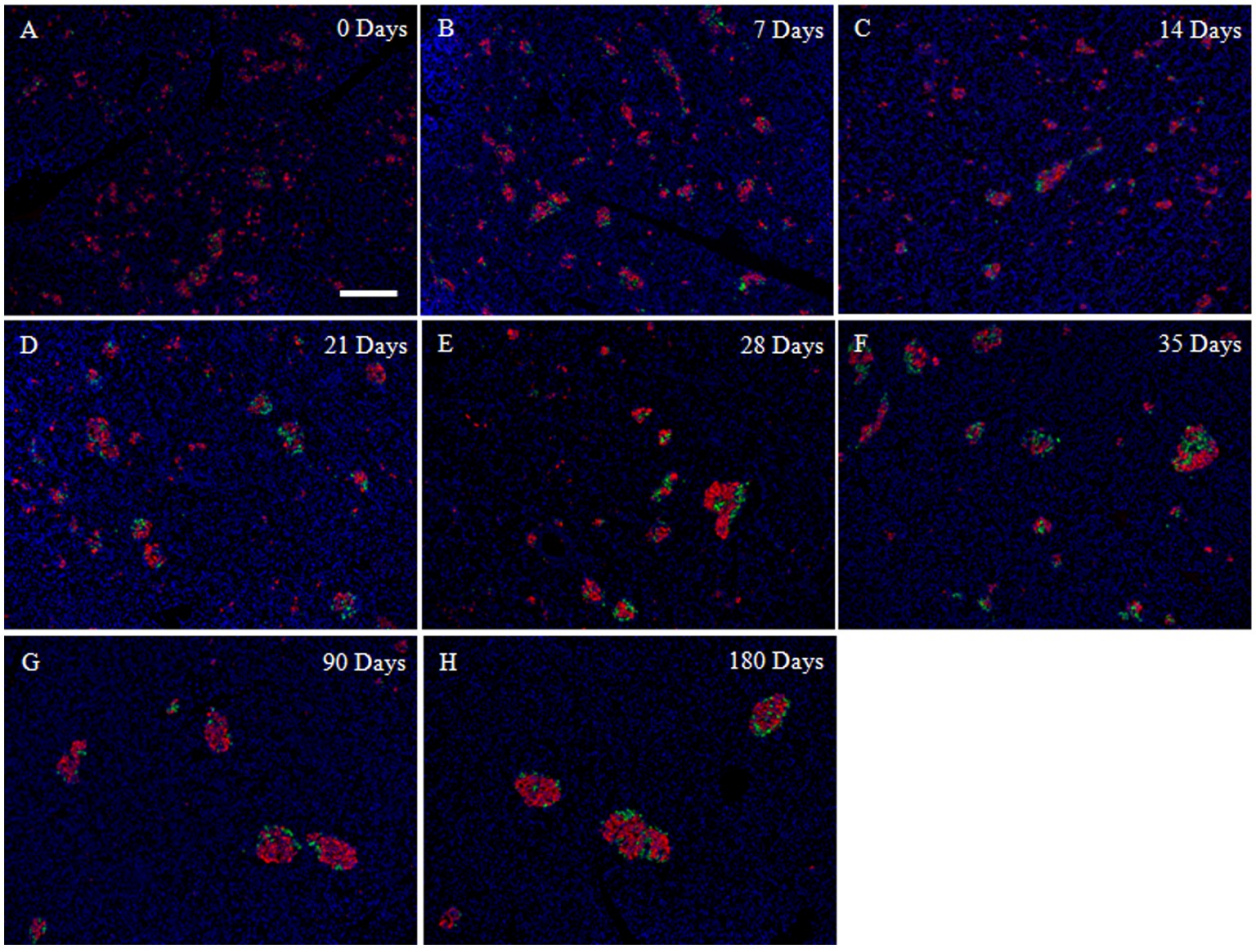
Spatial morphological changes in endocrine cell clustering during porcine pancreatic development from 0 to 180 days after birth. (A–H) The fluorescence micrographs show the expression of glucagon-positive cells (green) and insulin-positive cells (red). The nuclei are stained blue with Hoechst 33342. (A) 0 days, (B) 7 days, (C) 14 days, (D) 21 days, (E) 28 days, (F) 35 days, (G) 90 days, and (H) 180 days. The time after birth is represented as days. All of the tissue sections shown were obtained from the splenic lobe. All of the panels are at the same magnification. Scale bars: 200 μm.

At 0 days of age, the pancreas contained numerous cells with some endocrine cells scattered throughout the glands. Some ICCs were detected, but no islets were seen. In the SL, glucagon-positive cells were rarely seen (Fig 2A), and sections from the DLs contained very few glucagon-positive cells (data not shown). Low glucagon and insulin signals corresponded to the low frequencies of these cells.

From 7 days of age, insulin-positive cells were arranged in small groups and glucagon-positive cells were located not only within the cores, but also at the peripheries of the ICCs (Fig 2B). At 14 days of age, glucagon-positive cells were seen in the SL and DL, and these formed incomplete rings around the ICCs. The cytoarchitecture remained immature (Fig 2C). Sections from pigs that were 0–21 days of age showed that the endocrine cell clusters continued to undergo changes. From 21 days of age, the insulin-positive cells had started to become dense and compact, and islets appeared subsequently (Fig 2D). At 28 days of age, well-defined small islets had formed, they had become more prominent, and their numbers had increased dramatically (Fig 2E). At 35 days of age, the formation of small islets was similar to that observed at 90 days of age (Fig 2F and 2G), but the larger islets were not condensed. Greater numbers of islets were present, but single cells and small cell groups persisted. At 90 days of age, the overall pattern of insulin-containing tissue was similar to that found at 180 days (Fig 2G and 2H), and a few pairs of β-cells remained. The islets were clearly defined in the late stage group, and some were completely surrounded by collagen (data not shown). Most of the islets were oval, but some were triangular, dumbbell-shaped, and all the shapes in-between.

### Comparisons of the splenic and duodenal lobes regarding endocrine cell clustering during porcine pancreatic development

**Fig 3:**
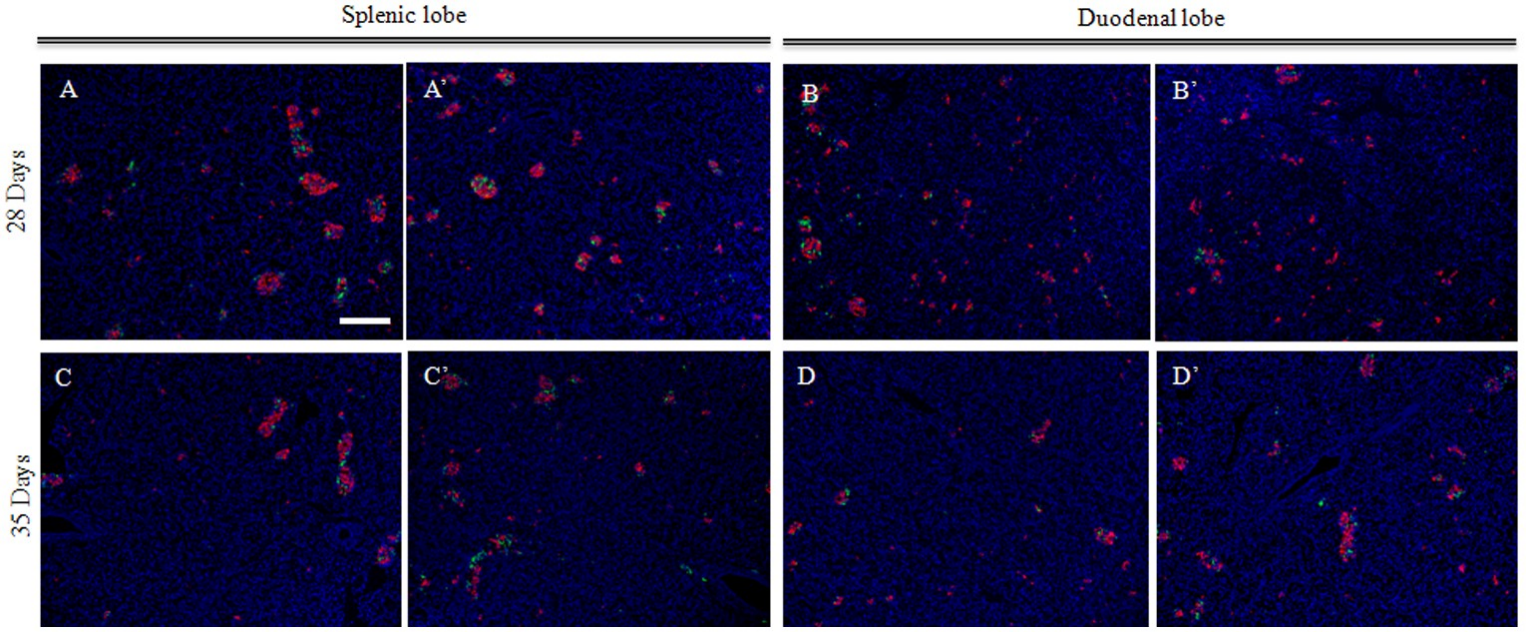
Comparisons between the splenic lobe (SL) and duodenal lobe (DL) in relation to endocrine cell clustering during porcine pancreatic development. (A–D, A’–D’) Morphological changes in the SL and DL in relation to endocrine cell clustering during porcine pancreatic development. The fluorescence micrographs show the expression of glucagon-positive cells (green) and insulin-positive cells (red). The nuclei are stained blue with Hoechst 33342. (A, A’) SL 28 days, (B, B’) DL 28 days, (C, C’) SL 35 days, and (D, D’) DL 35 days. The time after birth is represented as days. All of the panels are at the same magnification. Scale bars: 200 μm.

**Fig 4:**
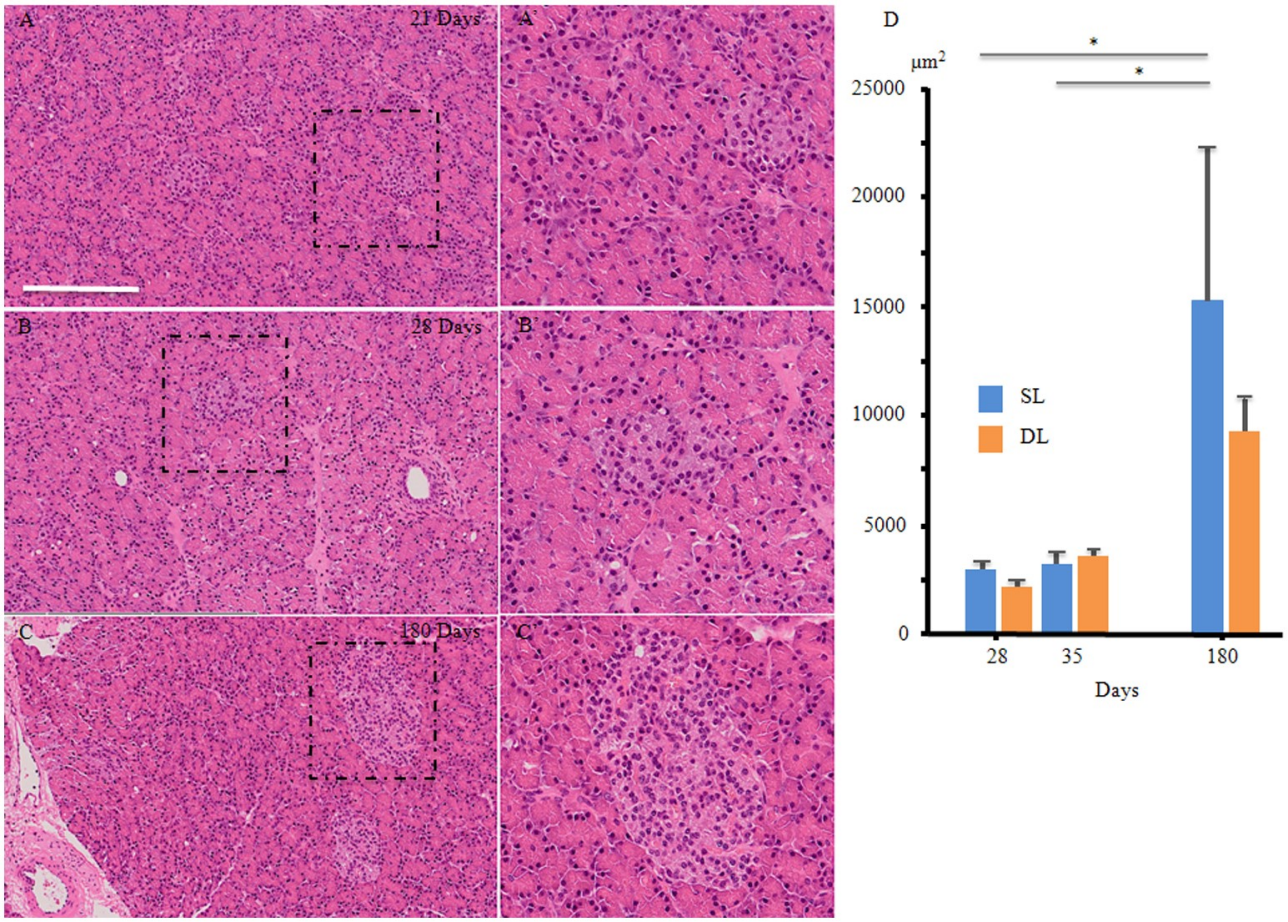
Chronological developmental changes and the areas of endocrine cell clustering during porcine pancreatic development. (A–C) Hematoxylin-eosin staining of the islets at each time point analyzed. (D) The islets’ areas were measured at each time point in both lobes. (A, A’) 21 days. The cell aggregates had started to compact and become dense, but the boundary lines are not clear. (B, B’) 28 days. Adult-like islet clusters were observed. Well-defined small islets were evident, but the gaps between the acinar cell structures and islets are narrow. (C, C’) 180 days. The islets in the adult pancreas showed denser cell populations with denser nuclear volumes overall. The time after birth is represented as days. All of the tissue sections shown were obtained from the splenic lobe. Panels A–C are at the same magnification, and panels A’–C’ are at the same magnification. Scale bars: 250 μm. (D) Islets’ areas at each time point. There were no significant differences between splenic lobe (SL) and duodenal lobe (DL) at each time point analyzed. The islets were counted in 3 randomly selected 1.65 mm^2^-areas, and sections were obtained from 3 pigs for each day studied. “Days” indicate days after birth. The data presented are the means atstandard errors of the means from independent experiments. The blue and orange bars indicate the SL and DL, respectively. *P < 0.05. SEM = standard error of the mean.

Compared with the endocrine cell clustering observed in the SL and DL at 28 days of age, the islets in the SL appeared more abundant than those in the DL. The islets were more loosely packed in the DL, and the β-cells remained prominent (Fig 3A, 3B, 3A’ and 3B’). No significant differences were evident between the SL and DL regarding the islets’ areas (Fig 4D), but the clusters were more dense in the SL than in the DL (Fig 3A, 3A’, 3B’ and 3B’). These differences did not exist at 35 days of age; the cytoarchitecture of the DL and SL was almost the same, and the islets’ areas were similar (Figs 3C, 3D, 3C’, 3D’ and 4D).

### Distributions of the endocrine cell cluster sizes during porcine pancreatic development

Table 2 presents data describing the distribution of the sizes of the clusters during porcine pancreas development. No significant differences were evident between 28 and 35 days of age regarding the short or long diameters. The short and long diameters of the islets were significantly greater at 180 days of age than those at 28 and 35 days of age (both *P* < 0.05). At 28 and 35 days of age, most of the islets’ diameters were < 100 μm (Table 2). The maximum islet diameter was 384.9 μm, and it was detected in the SL at 180 days of age.

**Table 2:** Distributions of the sizes of the endocrine cell clusters during porcine pancreatic development.

	SL		DL		SL + DL	
Age	Short diameter, μm	Long diameter, μm	Short diameter, μm	Long diameter, μm	Short diameter, μm	Long diameter, μm
28 days	52.7 ± 2.6*	75.5 ± 4.1*	45.9 ± 2.4*	67.2 ± 3.7*	49.7 ± 1.9*	71.9 ± 2.9*
35 days	50.0 ± 3.5^#^	75.8 ± 9.0 ^#^	54.7 ± 3.2^#^	84 ± 7.1^#^	52.7 ± 2.4^#^	80.6 ± 5.6^#^
180 days	109.0 ± 23.2*^#^	200.7 ± 44.1*^#^	84.3 ± 6.9*^#^	142.8 ± 13.8*^#^	91.4 ± 8.3*^#^	159.4 ± 16.3*^#^

SL = splenic lobe; DL = duodenal lobe.

*P < 0.0001,

^#^*P* < 0.0001.

The time after birth is represented as days.

### Comparisons of the areas in which the endocrine cells clustered during porcine pancreatic development

From 21 days of age, the dense and compact islets had started to appear (Fig 4A and 4A’). At 28 days of age, well-defined small islets had become more prominent. The gaps between the acinar cell structures and the islets were narrow (Fig 4B and 4B’).

The islets’ areas were significantly larger at 180 days of age (Fig 4C and 4C’) than those observed at 28 and 35 days of age in both lobes (both *P* < 0.05). No significant differences were evident between the SL and DL regarding the islets’ areas at any age (Fig 4D).

### Comparisons of the numbers of endocrine cells clustering during porcine pancreatic development

**Fig 5:**
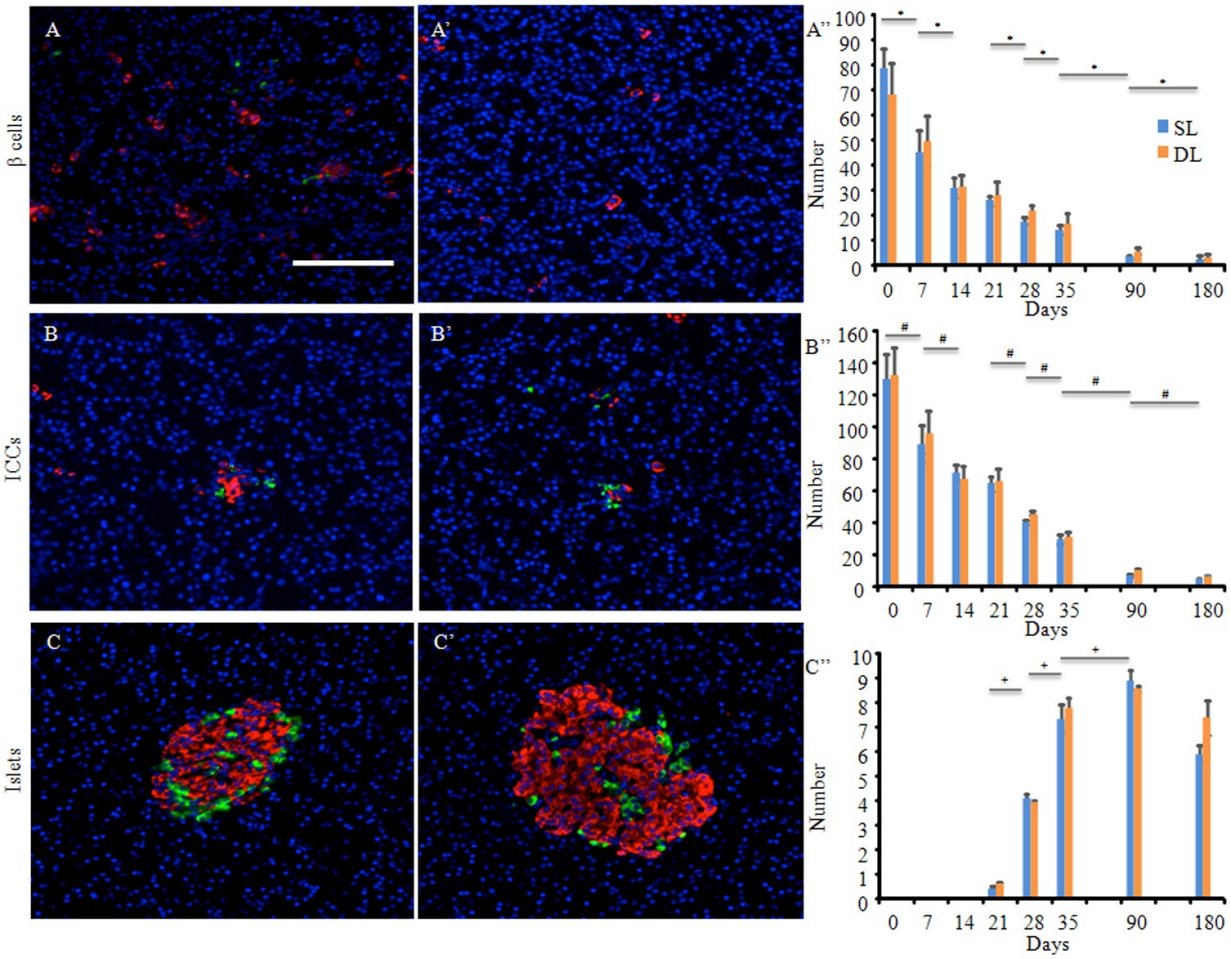
Comparisons of the pancreatic morphology and the numbers of β-cells, islet cell clusters (ICCs), and islets. (A–C, A’–C’) The fluorescence micrographs show the expression of glucagon-positive cells (green) and insulin-positive cells (red). The nuclei are stained blue with Hoechst 33342. Immunofluorescence analysis of (A, A’) β-cells at 7 days of age; the cells comprise only insulin-positive cells, (B, B’) ICCs, at 35 days of age; the ICCs are not compact, and consist of aggregates of glucagon- and insulin-positive cells of various sizes, and (C, C’) islets from the splenic lobe (SL) at 180 days of age; the islets comprise denser cell populations, and the edges of the aggregates are well defined. All of the tissue sections were obtained from the SL. All panels are at the same magnification. Scale bars: 100 μm. (A”–C”) Comparisons of the numbers of (A”) β-cells, (B”) ICCs, and (C”) islets in each lobe during porcine pancreatic development. There were no significant differences between the SL and duodenal lobe (DL) at each time point analyzed. The numbers of β-cells, ICCs, and islets were counted in 3 different randomly selected 1.65 mm^2^-areas from 3 pigs for each day assessed. The data presented are the means atstandard errors of the means. The blue and orange bars indicate the SL and DL, respectively. SL versus DL: all *P* < 0.05. SEM = standard error of the mean.

The numbers of β-cells (Fig 5A and 5A’) and ICCs (Fig 5B and 5B’) decreased significantly chronologically, except between 14 and 21 days of age (Fig 5A” and 5B”). There were no significant differences between the SL and DL regarding the numbers of β-cells and ICCs at any age. The numbers of islets (Fig 5C and 5C’) increased dramatically from 21 days of age (Fig 5C”). There were no significant differences between the SL and DL regarding the numbers of islets at any age, and their numbers increased significantly until 90 days of age. At around 35 days of age, a greater number of islets were present, although β- cells and ICCs were still observed (Fig 5A”, 5B” and 5C”). Similar observations were recorded, even at the late stage (Fig 5A”and 5C”).

### Gene regulatory networks underlying endocrine cell clustering for differentiation and maturation during porcine pancreatic development

**Fig 6:**
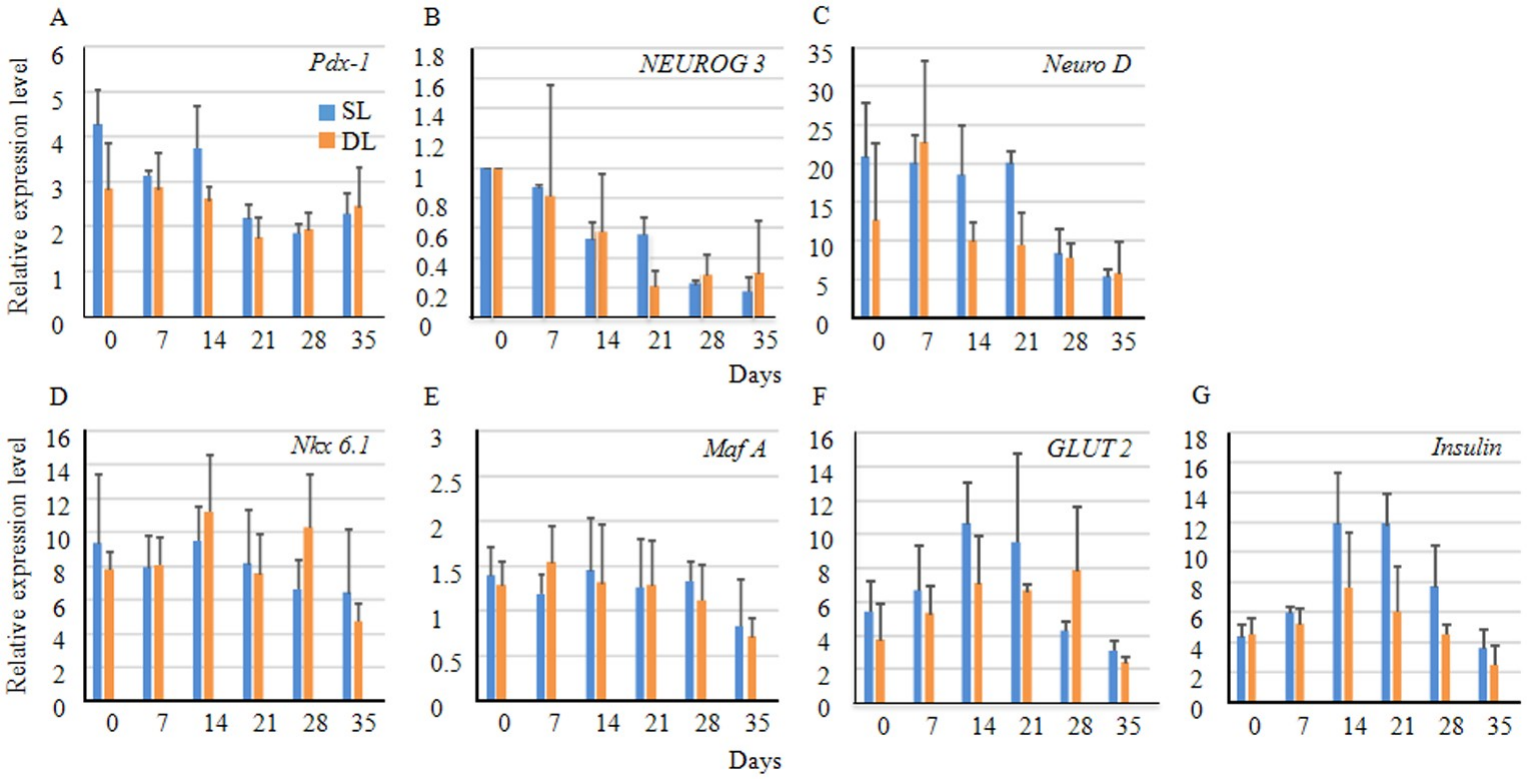
Comparisons of the messenger ribonucleic acid (mRNA) levels of selected genes. (A–G) Gene regulatory networks underlying endocrine cell clustering relating to differentiation and maturation during porcine pancreatic development. The mRNA levels of the β-cell-related genes (A) *pancreatic and duodenal homeobox 1* (*PDX1*), (B) *neurogenin-3* (*NGN3)*, (C) *neuronal differentiation 1* (*NEUROD1*), (D) *NK6 homeobox 1* (*NKX 6–1*), *v-maf avian musculoaponeurotic fibrosarcoma oncogene homolog A* (*MAFA*), (F) *glucose transporter type 2* (*GLUT2*), and (G) *insulin (INS)* were determined using real-time polymerase chain reactions. The mRNA levels were normalized to those of *actin* actin gamma 1(*ACTG1*). Except for *NGN3*, the normalized values were compared with those in the pancreas at 180 days after birth. For the *NGN3* expression, samples from 0 days after birth were used for the normalization. The expression levels were determined based on the averages from triplicate assays. The data presented are the means T standard errors of the means from 3 independent experiments. The blue and orange bars indicate the splenic lobe (SL) and duodenal lobe (DL), respectively. SEM = standard error of the mean.

All of the transcription factor genes, except *NGN3*, were expressed constantly and at random (Fig 6). The expression of all of the genes was higher in the juvenile than in the adult porcine pancreases, and it tended to be higher in the SL than in the DL. The expression of *NGN3* decreased dramatically with time, and it was almost indistinct at 180 days of age. Like the expression of *NGN3*, the expression of *neuronal differentiation 1* fluctuated. The expression patterns of the *glucose transporter 2* (*GLUT2*) and *insulin (INS)* genes were similar, and both were abundant from 0 to 21 days of age. The expression of all of the genes tended to decrease with time.

## Discussion and conclusion

In this study, porcine pancreatic development was categorized into 3 stages, namely, early (0–21 days of age), middle (28–35 days of age), and late (≥ 90 days of age). Alumets et al. demonstrated that until 10–13 days after birth, the cells did not cluster in small islets, and that many cells remained scattered within the exocrine parenchyma [34]. These findings are similar to those from our study, and their results support our definitions of ICCs and islets, because the islets did not appear until 21 days. At 21 days of age, dramatic changes in the clusters were observed, and, at that age, we detected a second developmental change among the maturing islets. The change may have been influenced by weaning, which occurs at around 21–28 days of age among pigs, and islet development is associated with this age [35].

In mice, islet formation occurs just before birth [19], but islets appear in human fetuses at 11–15 weeks, and they develop into relatively large structures several weeks before birth [22]. In the pigs, the islets did not form until the middle stage in this study, and mature islets (large, dense cell aggregates that comprised glucagon- and insulin-positive cells with well-defined edges) were not seen until the late stage. Thus, the islets develop at different speeds in different mammals. In addition, islet cytoarchitecture differs among animal species [22, 23, 36]. For example, while rodent islets are characterized by a predominance of insulin-producing cells in the cores of the clusters and scarce α-, δ-, and pancreatic polypeptide cells at the peripheries, human islets contain α- and β-cells that are closely associated with each other throughout the clusters [37]. The distribution of the islets in the pigs was established by 28–35 days of age, and glucagon-positive cells were located within the cores of the islets as well as around their peripheries at all ages. Our study’s findings indicate that porcine islets more closely resemble human islets than rodent islets.

Another difference was observed regarding the distributions of the islets between the SL and DL of the pancreas. Sections of porcine pancreas from the SL and DL were studied, because they originate from separate primordia and their morphologies differ [5]. Some animals, including mice, rats, dogs, and humans have more islets in the SL than in the DL [38, 39], but in the pigs, there were no differences between the lobes.

Glucagon-positive cells were seen at 0 days of age. In addition, the clusters were denser and the glucagon signal was higher in the SL than in the DL at 28 days of age. Interestingly, Lyttle et al., demonstrated changes in the glucagon-positive cell mass that coincided with significant increases in the numbers of Ki67^+^/glucagon^+^ cells. The rapid expansion of glucagon-positive cells is mainly caused by the replication of and changes in the β-cell mass that correlate with increases in the pancreatic insulin content and secretion [23]. Thus, the appearance of and changes in glucagon-positive cells indicate islet development. Jay et al. showed that glucagon-positive cells were the second-most predominant cell group at all ages in the SL and that they occurred less frequently in the DL of the pancreas [5]; these findings concur with our results.

Regarding islet diameter, White et al. undertook a computerized image analysis study, and their findings showed that the mean diameters of the islets in adult and juvenile pigs were 80 μm and 87 μm, respectively [40]. Other researchers have shown islet diameters to be between 49 μm and 100 μm [39, 41]. Islets have different shapes, and we measured the short and long diameters of each islet (S2 Fig), and the results concurred with those from previous studies. According to Jay et al., the most significant difference regarding the diameter of the islets was evident at 35 days of age. By 84 and 168 days of age, the greatest volume density within the SL of the pancreas comprised islets with diameters that ranged from 50 μm to 149 μm [5]. Importantly, they also showed that the islets comprised the greatest numbers of cell groups and a substantial proportion of the volume density, especially at the youngest age of 35 days; the results from our study align with these findings.

In rodents, some genes show characteristic expression patterns. For example, *neurogenin-3* expression suggests that cells have the progenitor characteristics of β-cells [20, 26– 28], and its expression ceases after the secondary transition [25, 28]. We found that the development of the pancreas in pigs was slow, so the duration of *NGN3* expression appeared to be extended. The numbers of β-cells and ICCs decreased over time, which coincided with a decrease in *NGN3* expression. β-cells and/or ICCs in pigs may possess the progenitor characteristics of β-cells, and this should be investigated during the next stages of our research. In humans, unlike mice, the expression of *NGN3* is not synchronized with that of endocrine differentiation factors following the secondary transition [23, 29]. Other transcription factors in this study were expressed constantly and at random. The expression of all of the genes investigated was higher in the juvenile pancreas than in the adult pancreas. Transitional events may occur, but at multiple foci and without the same temporal coincidence as those in mice or humans.

Taking these findings together, we suggest that pancreatic islets isolated from pigs aged around 35 days could be used as xenotransplants. Compared with the late-stage islets, the islets’ shapes were similar, and the number of islets was adequate at around 35 days of age. Further, previous differences in the islets’ cytoarchitecture between the SL and DL were negligible in pigs aged 35 days. Isolating islets from pigs aged around 35 days would be advantageous because large isolated islets often succumb to central necrosis caused by the inadequate diffusion of oxygen and nutrients, which may lead to graft failure after transplantation before the graft becomes vascularized. As most of the islets in pigs aged around 35 days are < 100 μm, they may be better suited to transplantation, because essential nutrients should diffuse more easily [42]. Young adult porcine islets release higher levels of insulin in response to glucose [43]. Our analyses of the qPCR results showed that the expression of genes in the juvenile porcine pancreas, including the *GLUT2* and *INS* genes, was higher than that observed in the adult porcine pancreas. Next, we will investigate the appropriate method for isolating islets from pigs aged 35 days because this differs from the method used to isolate islets from adult humans. Islets in pigs aged around 35 days are smaller than those in adult pigs and humans, and the Ricordi isolation method will not be suitable [44]. We suggest that 35 days of age is suitable for porcine islet isolation, but there are a number of questions. The optimal age of the islet from a porcine source is of major importance to the future of islet xenotransplantation. In our study, we have used only LWD pig strain and both sexes of pigs. In adult pigs, it has been known that strain influences the islet yield [41, 45– 49]. However, Prabhakaran S et al and Nagaraju S et al concluded that there is no clear information concerning the yield in relation to the species of fetal/neonatal donor pigs [50, 51]. The confirmation whether our study could extrapolate to other strains would be addressed in our future study. Regarding the sex chosen, retired female breeders of large size certainly have been favored for the high yield and good compact morphology of the islets [52– 54]; however, both male and female pigs have been used as donors [55, 56] and there is no convincing information concerning the yield in relation to the sex of neonatal donor pigs.

The expression patterns of the glucose transporter 2 (GLUT2) and insulin (INS) genes were both abundant from 0 to 21 days of age. However, we do not know how the higher gene expressions influence islet isolation and transplantation. This would be investigated after isolation of the islets from pigs aged 35 days and transplantation.

Further studies are required to assess whether isolated islets at 35 days of age will function as expected.

## Supporting information

S1 Fig Architectural change in the porcine pancreas during development. (A–D, A’–D’) Hematoxylin and eosin staining shows changes in the pancreas structure at each time point. The histological analyses revealed progressive developmental changes in the pancreas. (A, A’) 14 days. The lobule structure of the pancreas is evident, but not elaborate. The duct- and vessel-like structures were thin and immature. (B, B’) 21 days. The structures of the duct- and vessel-like structures resembled those at 14 days of age. (C, C’) 28 days. Significant changes in the tissue architecture were noted. The circumferences of the duct- and vessel-like structures had thickened as a consequence of connective tissue surrounding these structures to resemble the adult configuration (D, D’) 180 days. The time after birth is represented as days. All of the tissue sections were obtained from the splenic lobe. All of the panels are at the same magnification. Scale bars: 250 μm.
(TIF)

S2 Fig Measurement of the islets. Islets from the pancreas sections analyzed in Fig 5 are shown according to their apparent areas. After immunofluorescence, the glucagon- and insulin-positive areas were demarcated and measured using a computer system (Cosmos32 Library, Tokyo, Japan).
(TIF)

## Abbreviations

DL: duodenal lobe
*GLUT2*: *glucose transporter 2*
ICCs: Islet cell clusters
*INS*: *insulin*
*MAFA*: *v-maf avian musculoaponeurotic fibrosarcoma oncogene homolog A*
*NEUROD1*: *neuronal differentiation 1*
*NGN3*: *neurogenin-3*
*NKX6-1*: *NK6 homeobox 1*
*PDX1*: *pancreas duodenum homeobox -1*
PERVs: porcine endogenous retroviruses
q-PCR: quantitative Reverse Transcription Polymerase Chain Reaction
SL: Splenic lobe
