# Supplementary figures and images for: Distributions of endocrine cell clusters during porcine pancreatic development
Source: PLoS One. 2019 May 10;14(5):e0216254. doi: 10.1371/journal.pone.0216254 (PMC6510474; doi:10.1371/journal.pone.0216254)

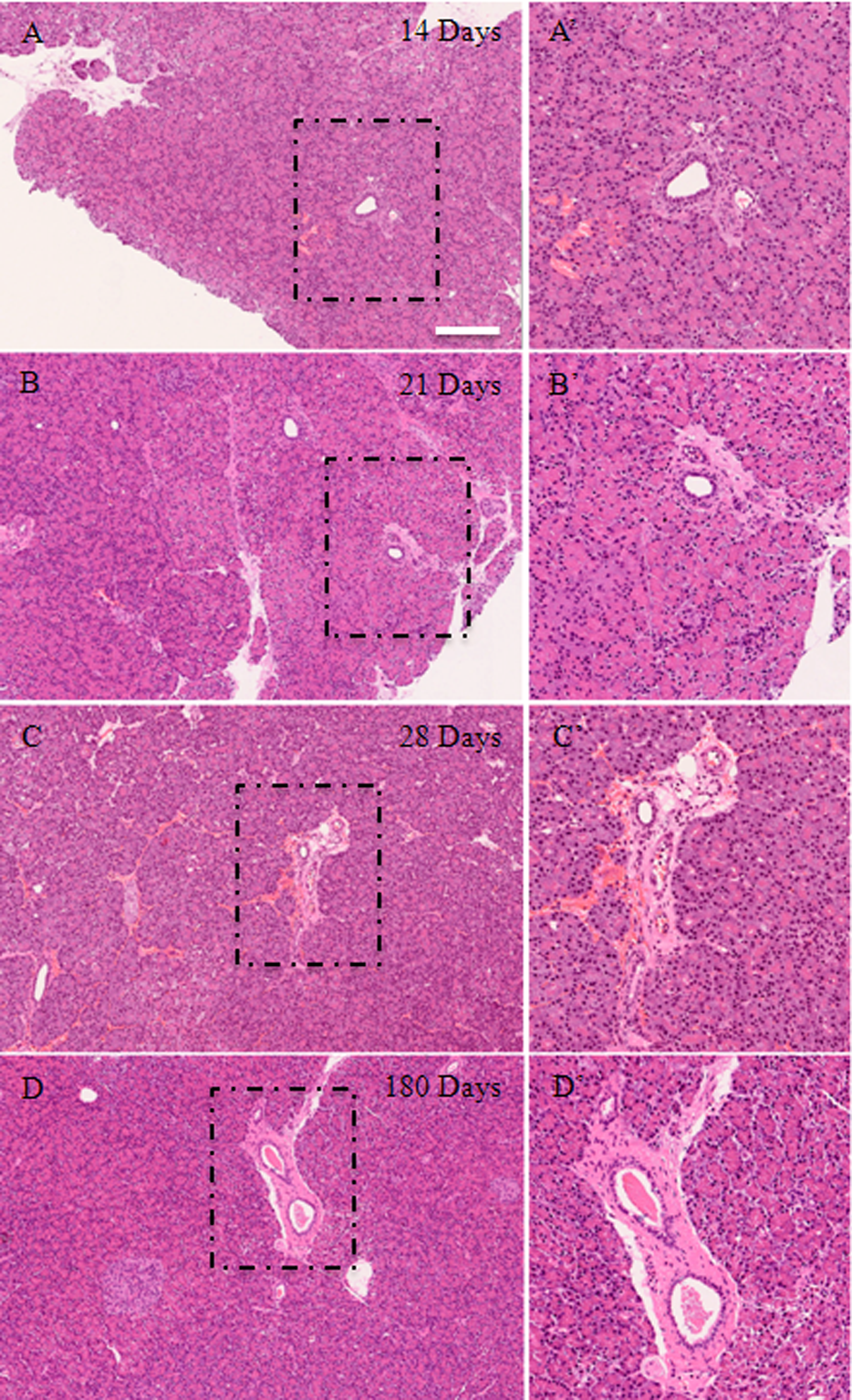

Supplement: S1 Fig — (A–D, A’–D’) Hematoxylin and eosin staining shows changes in the pancreas structure at each time point. The histological analyses revealed progressive developmental changes in the pancreas. (A, A’) 14 days. The lobule structure of the pancreas is evident, but not elaborate. The duct- and vessel-like structures were thin and immature. (B, B’) 21 days. The structures of the duct- and vessel-like structures resembled those at 14 days of age. (C, C’) 28 days. Significant changes in the tissue architecture were noted. The circumferences of the duct- and vessel-like structures had thickened as a consequence of connective tissue surrounding these structures to resemble the adult configuration (D, D’) 180 days. The time after birth is represented as days. All of the tissue sections were obtained from the splenic lobe. All of the panels are at the same magnification. Scale bars: 250 μm. (TIF) [file pone.0216254.s001.tif]

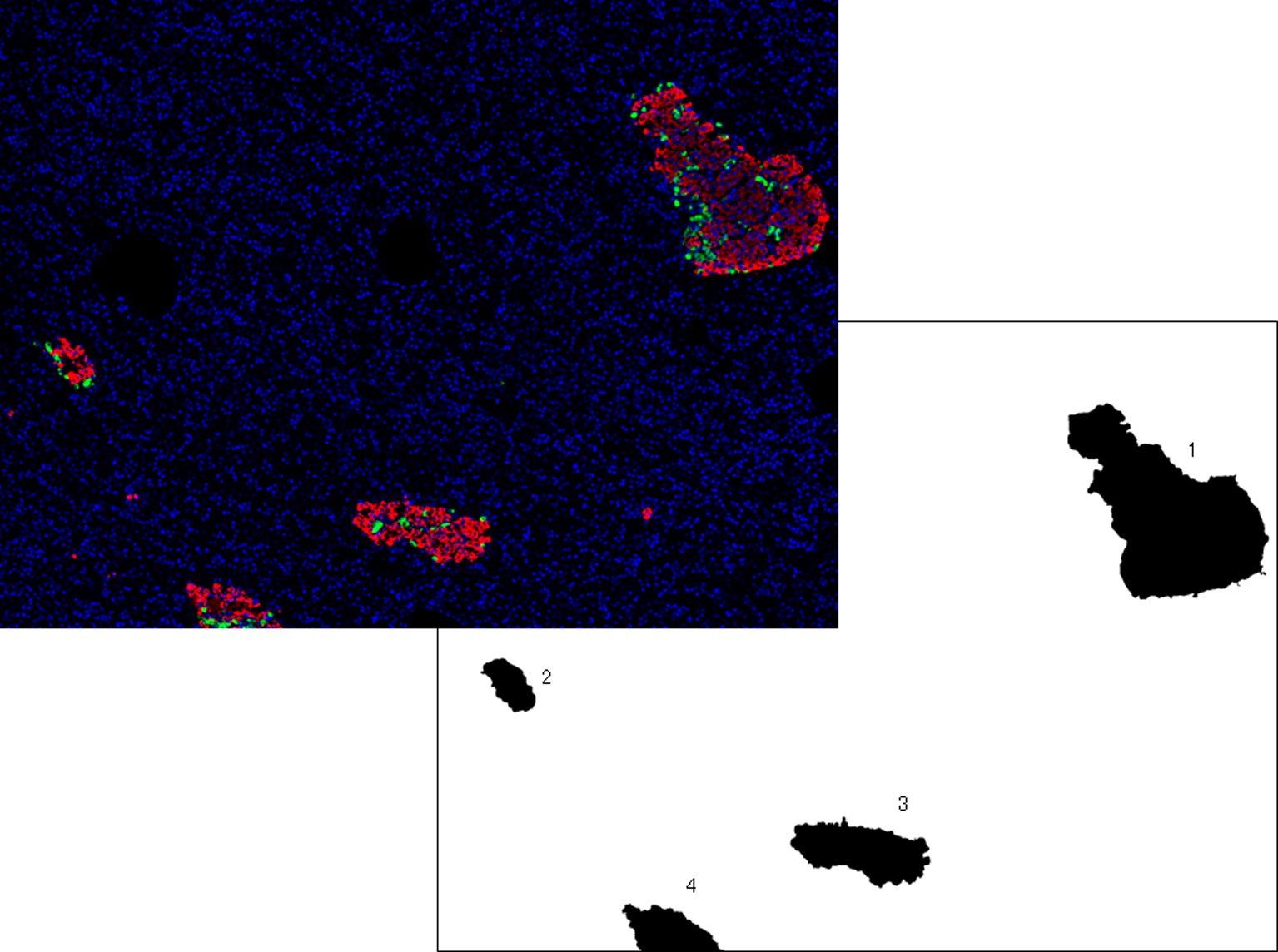

Supplement: S2 Fig — Islets from the pancreas sections analyzed in Fig 5 are shown according to their apparent areas. After immunofluorescence, the glucagon- and insulin-positive areas were demarcated and measured using a computer system (Cosmos32 Library, Tokyo, Japan). (TIF) [file pone.0216254.s002.tif]
